# Effect of β-Estradiol on Adipogenesis in a 3T3-L1 Cell Model of Prelamin A Accumulation

**DOI:** 10.3390/ijms25021282

**Published:** 2024-01-20

**Authors:** Silvia Cobelo-Gómez, Sofía Sánchez-Iglesias, Antía Fernández-Pombo, David Araújo-Vilar

**Affiliations:** Thyroid and Metabolic Diseases Unit (U.E.T.eM.), Department of Psychiatry, Radiology, Public Health, Nursing and Medicine, Center for Research in Molecular Medicine and Chronic Diseases (CIMUS)-IDIS, University of Santiago de Compostela, 15782 Santiago de Compostela, Spain; silviacobelog@gmail.com (S.C.-G.); sofia.sanchez@usc.es (S.S.-I.); antiafpombo@gmail.com (A.F.-P.)

**Keywords:** *LMNA*, prelamin A, adipogenesis, FTI-277, AFCMe, 17-β estradiol, familial partial lipodystrophy type 2

## Abstract

The accumulation of farnesylated prelamin A has been suggested as one of the mechanisms responsible for the loss of fat in type 2 familial partial lipodystrophy due to variants in the *LMNA* gene. In this rare disease, fat loss appears in women after puberty, affecting sex-hormone-dependent anatomical areas. This study investigated the impact of 17-β-estradiol on adipogenesis in murine preadipocytes subjected to a pharmacologically induced accumulation of farnesylated and non-farnesylated prelamin A. To induce the accumulation of non-farnesylated or farnesylated prelamin A, 3T3-L1 cells were treated with the farnesyltransferase inhibitor 277 or the methyltransferase inhibitor N-acetyl-S-farnesyl-l-cysteine methylester. Subsequently, the cells were induced to undergo adipocyte differentiation in the presence or absence of 17-β-estradiol. Prelamin A accumulation was assessed through immunofluorescence, while real-time PCR and Western blot techniques were used to quantify several adipogenic genes and evaluate protein levels, respectively. The results showed that 17-β-estradiol increased adipogenesis, although the combination of this hormone plus farnesylated prelamin A led to a reduction in the number of mature adipocytes and the expression of the different genes involved in adipogenesis. In conclusion, the influence of farnesylated prelamin A accumulation on adipogenesis manifested only in the presence of estradiol. These in vitro findings suggest a potential mechanism that could explain the characteristic phenotype in women suffering type 2 familial partial lipodystrophy.

## 1. Introduction

Type 2 familial partial lipodystrophy (FPLD2; OMIM #151660) is characterized by the loss of subcutaneous adipose tissue in the limbs and buttocks, accumulation of fat in the neck and face, and a predisposition to insulin resistance, leading to complications such as diabetes, dyslipidemia, liver steatosis, and increased risk of coronary heart disease [1,2].

FPLD2 results from variants in the *LMNA* gene [3], which codes for several spliced proteins, include lamin A, lamin C, lamin C2, and lamin AΔ10. Lamin A, a key component of the nuclear lamina, is formed from post-translational modifications of a precursor protein, prelamin A [4]. Prelamin A is initially modified by a farnesyltransferase that adds a 15-carbon farnesyl isoprenoid to the carboxyl-terminal cysteine. Farnesylated prelamin A is then modified by a prenyl-CAAX-specific endoprotease (AAX = aliphatic-aliphatic-any amino acid), known as RAS-converting enzyme 1, which removes the last three carboxyl-terminal amino acids. ZMPSTE24, the enzyme required for the last proteolytic step, has also been proposed to perform this cleavage reaction. This prelamin A intermediate then becomes the substrate for a second ER-resident enzyme, isoprenylcysteine carboxyl methyl-transferase, which methylesterifies the carboxyl-terminal farnesylcysteine. Lastly, farnesylated prelamin A is processed by ZMPSTE24, which cleaves the carboxyl-terminal 15 amino acids, releasing the mature, non-farnesylated lamin A [5]. 

Several studies, both in vitro [6] and ex vivo [7], have demonstrated prelamin A accumulation in the nuclei of cells from subjects suffering FPLD2. Furthermore, Capanni et al. found that prelamin A co-localized with the adipocyte transcription factor sterol regulatory element binding protein 1 (SREBP1). Using co-immunoprecipitation experiments, these authors demonstrated an in vivo interaction between SREBP1 and prelamin A, suggesting that prelamin A sequesters SREBP1 at the nuclear rim, thus decreasing the pool of active SREBP1 that normally activates PPARG and causing an impairment of preadipocyte differentiation [8].

On the other hand, our group [9] has reported that prelamin A was also present in the adipose tissue of lipohypertrophic areas, albeit in a lower proportion than in lipoatrophic areas, in a patient bearing the classical p.(R428W) variant in *LMNA*. This suggests that prelamin A accumulation could be a necessary but not sufficient condition for the impairment of the adipogenic process [10,11,12].

The relationship between the distribution of body fat between sexes and sexual hormones (androgens and estrogens) plays an important role in the generation and distribution of adipose tissue with a balance between the levels of circulating estrogens and testosterone being of apparent importance [13]. On the other hand, to the best of our knowledge, there are no studies on a putative relationship between lamin A/C, estrogens and adipogenesis. However, in patients suffering FLPD2, it is remarkable that the lipodystrophic phenotype frequently appears around puberty in women but not in men [1,2]. Thus, given that fat loss appears in women after puberty, affecting anatomical areas dependent on sex hormones (limbs and buttocks) [14] and, on the other hand, many women adopt an android pattern of fat distribution after menopause [10,11,12], it is appealing to hypothesize that female sexual hormones, with 17 β-estradiol being the most biologically active [15], should play some role in the pathogenetic mechanism responsible for the loss of adipose tissue in these patients.

## 2. Results

### 2.1. Assessment of Adipogenic Differentiation

**Figure 1:**
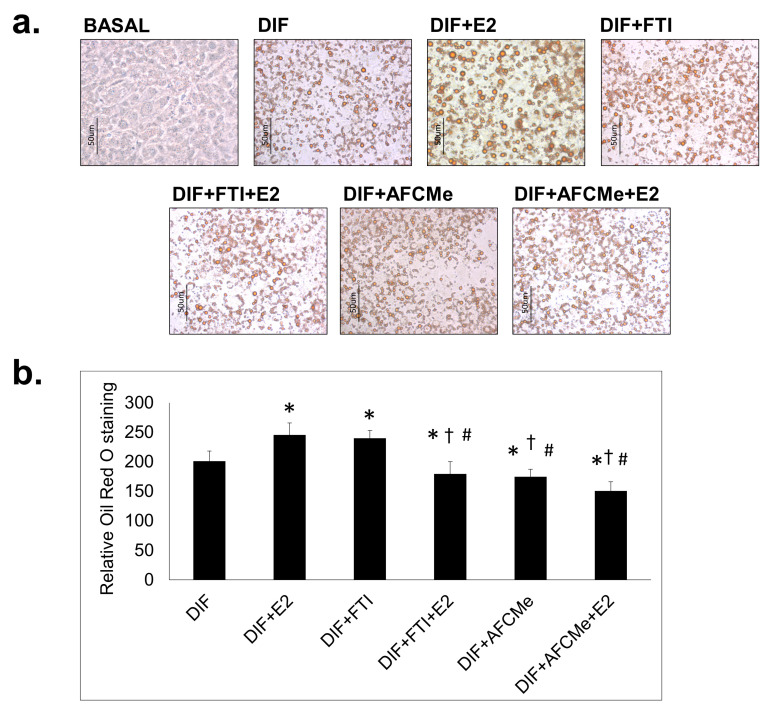
*Oil Red O* staining of treated and untreated 3T3-L1 preadipocytes. 3T3-L1 cells were induced to differentiate (DIF) in 6-well plates for eight days in the absence or presence of β-estradiol (E2) together or not with FTI-277 or AFCMe. (**a**) Representative images (40×) of the *Oil Red O* staining are shown for each treatment group. The scale bar represents 50 μm. (**b**) Quantification of three images in random field for each of the conditions of the three independent experiments. *: *p* < 0.05 compared to DIF; †: *p* < 0.05 compared to DIF + E2; #: *p* < 0.05 compared to DIF + FTI. Colorimetric quantification was performed with the DigitalColor Meter tool (Macintosh, Apple Inc., Cupertino, CA, USA).

Adipogenic differentiation was evaluated through *Oil Red O* staining of intracellular lipids and observation under bright-field microscopy. The traditional adipocyte differentiation cocktail, supplemented by pioglitazone, resulted in the accumulation of lipid droplets in adipocytes. Treatment with β-estradiol further enhanced lipid accumulation in these cells (Figure 1). When treated only with inhibitors of prelamin A maturation, FTI-277 increased the number of mature adipocytes, while AFCMe reduced their quantity. However, the combined treatment of these inhibitors with β-estradiol significantly decreased the number of mature adipocytes (Figure 1b).

### 2.2. Prelamin A Accumulation after Drug Treatment

**Figure 2:**
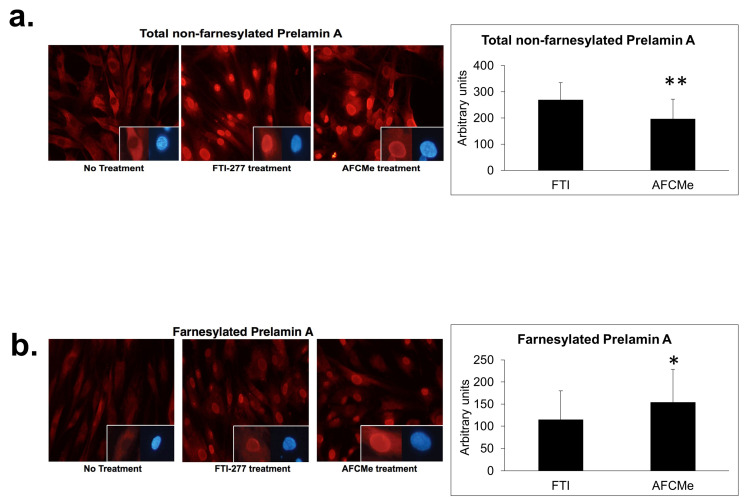
Nuclear localization of prelamin A and farnesylated prelamin A. Nuclear localization of full-length non-farnesylated prelamin A (**a**) or carboxymethyl-farnesylated prelamin A (**b**) in control human preadipocytes. At the bottom right of the photographs, nuclei are shown labeled with anti-prelamin A antibodies (red) and counterstained with DAPI (blue) on the right side. Cells were visualized with an Olympus IX51 microscope (20× objective, Olympus Corporation, Tokyo, Japan). Colorimetric quantification was performed with the DigitalColor Meter tool (Macintosh, Apple Inc., Cupertino, CA, USA). Data are mean ± SD, *: *p* < 0.01, **: *p* < 0.01.

Human preadipocytes were used to determine prelamin A accumulation after FTI-277 or AFCMe treatment [7]. It is noteworthy that carboxymethyl-farnesylated prelamin A is selectively detected using ANT0046 antibody, while antibody ANT0045 does not bind this prelamin A form. Using immunofluorescence microscopy, it was found that FTI-277 and AFCMe treatments impede proper maturation. FTI-277 induced migration and notable nuclear accumulation of full-length non-farnesylated prelamin A in the nuclei of preadipocytes (Figure 2a), while AFCMe inhibition of prelamin A maturation led to a significant accumulation of the farnesylated form (Figure 2b).

### 2.3. Effect of β-Estradiol and/or Inhibitors of Prelamin A Maturation in Adipogenic Gene Expression

**Figure 3:**
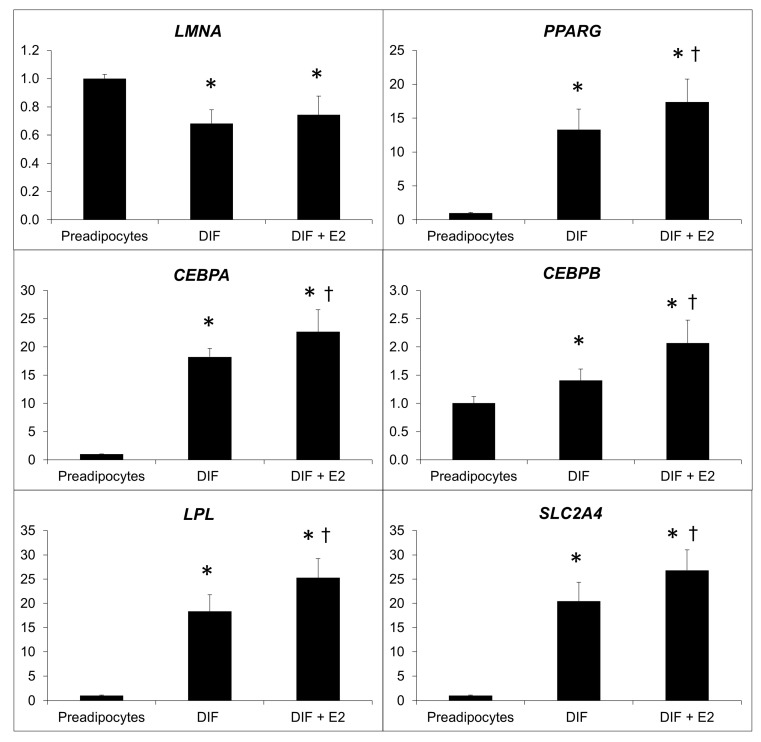
Effect of the treatment of β-estradiol on the relative expression of adipogenic genes. Relative expression of *LMNA*, *PPARG*, *CEBPA*, *CEBPB*, *LPL*, and *SLC2A4*, normalized to the *18S* gene before and after β-estradiol (E2) treatment. *: *p* < 0.05 vs. preadipocytes, †: *p* < 0.05 vs. DIF. Analysis of four independent experiments in duplicate.

The differentiation of preadipocytes into mature adipocytes resulted in a notable decrease in the expression of the *LMNA* gene. Treatment with estradiol significantly increased the expression of key genes associated with adipocyte differentiation and maturation (Figure 3).

**Figure 4:**
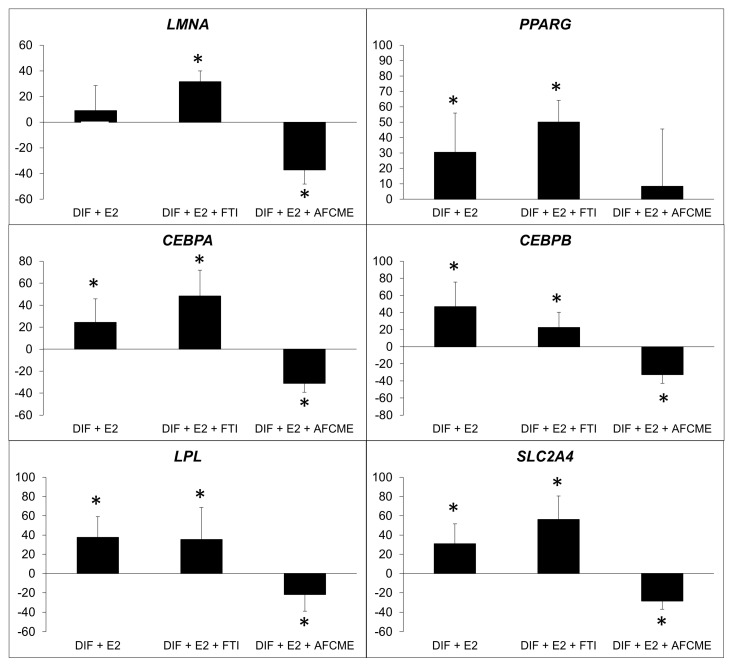
Percentage of change in expression of adipogenic genes. Percentage of change in expression of *LMNA*, *PPARG*, *CEBPA*, *CEBPB*, *LPL* and *SLC2A4*, which refer to the relative expression of adipocytes treated only with β-estradiol (E2) or also treated with FTI-277 or AFCMe, and normalized to the *18S* gene. *: *p* < 0.01 refers to differentiated adipocytes with no treatment. Four independent experiments were analyzed in duplicate.

Combined treatment with β-estradiol and inhibitors of prelamin A maturation revealed that the accumulation of farnesylated prelamin A reduces the effect of this hormone on the expression of the examined adipogenes. In contrast, the accumulation of total prelamin A (non-farnesylated) significantly increased the adipogenic gene expression in cells treated with β-estradiol (Figure 4).

### 2.4. Effects of Processing Inhibitors in Adipogenic Protein Evaluation

**Figure 5:**
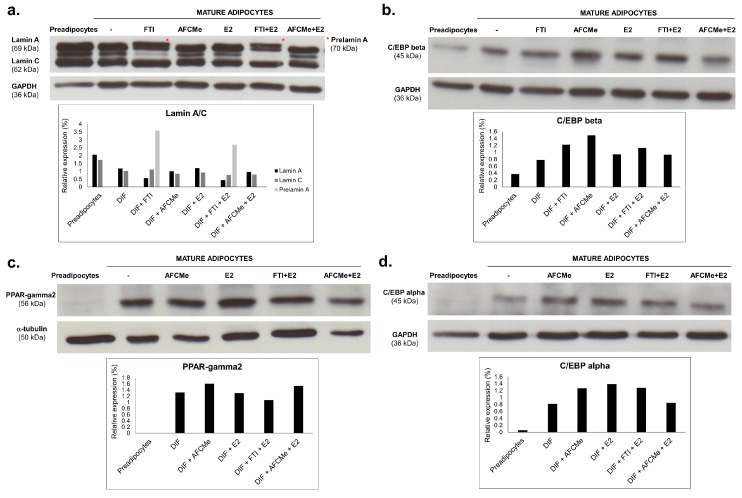
Effect of processing inhibitors in drug-treated 3T3-L1 adipocytes. Western blot analysis and quantification of lamin A/C and prelamin A (*) (**a**), C/EBP beta (**b**), PPAR-gamma2 (**c**) or C/EBP alpha (**d**) in adipocytes treated with β-estradiol and/or with FTI-277 or AFCMe, as well as in control 3T3-L1 preadipocytes and mature adipocytes. Quantification from two independent experiments.

To assess the impact of processing inhibitors, Western blot analysis was employed to quantify lamin A/C, prelamin A, CEBPbeta, CEBPalpha and PPARgamma2 levels (Figure 5). Prelamin A was undetectable in untreated mature adipocytes and following AFCMe treatments, while it was increased in cells subjected to FTI-277 treatments. Interestingly, an intermediate band was detected between lamin A and C, suggesting the presence of progerin as the intensity is decreased in cells treated with FTI-277. Consistent with gene expression findings, protein level analysis revealed that the amounts of lamin A and C in mature adipocytes were lower than in preadipocytes (Figure 5a). Regarding adipogenic proteins, adipocyte differentiation correlated with a higher level of PPAR-gamma2, C/EBP beta and C/EBP alpha. However, the pro-adipogenic effect of estradiol was evident only for C/EBP beta and C/EBP alpha. On the other hand, although the visual inspection of band intensity may suggest that combined treatment with estradiol plus AFCMe reduced the amount C/EBP beta, C/EBP alpha and PPAR-gamma2, densiometric analysis revealed that this reduction was observed only for C/EBP alpha (Figure 5b–d).

## 3. Discussion

The primary objective of this study was to investigate the effect of 17-β-estradiol on the adipogenesis of murine 3T3-L1 preadipocytes, both in the presence or absence of prelamin A maturation inhibitors, thus establishing an in vitro model of prelamin A accumulation. Differences were observed based on exposure to β-estradiol and the use of FTI-277 and AFCMe as inhibitors.

Prelamin A accumulation in the nuclei of cells has been reported to be involved in the adipogenic process [8]. Our study, consistent with earlier research, identified the presence of both farnesylated and full-length non-farnesylated prelamin A in the nucleus, suggesting potential toxicity due to peripheral accumulation [16,17]. However, as proposed by Araújo-Vilar et al., this would not be sufficient for the impairment of the adipogenic process [7].

It is well known that sex hormones, such as estrogen, act on adipogenesis influencing body fat distribution [18] and that adipogenesis involves numerous factors which allow the differentiation of preadipocytes to mature adipocytes [19]. Some of these factors are PPARgamma, members of the CCAAT/enhancer-binding proteins (C/EBP) family, as well as Krüppel-like factors, the signaling pathway of the Wingless protein, AP-1 and SREBP-1c [20].

As previously commented, no direct studies establishing a relationship among estrogens, lamin A and adipogenesis have been published. However, a relationship between estrogens and lamin A has been reported, which is mainly in relation to hormone-dependent malignancies [21,22]. In addition, treatment with fulvestrant, an estrogen receptor antagonist, inhibits osteoblast differentiation and increases *LMNA* expression and lamin A/C levels [23]. Furthermore, Elisa Schena et al. [24] have recently shown that the mineralocorticoid receptor is a new player in FPLD2. Interestingly, it has been reported that the G protein-coupled estrogen receptor (GPER) is a newly discovered aldosterone receptor, which is proposed to mediate the non-genomic pathways of aldosterone while interacting with estrogen [25]. Although this is not proof of the putative role of estrogens in modulating lamin A function on adipose tissue, it probably paves the way for future studies on this topic.

As expected, the differentiation cocktail led to the complete adipocyte differentiation of 3T3-L1 preadipocytes, showing an accumulation of lipid droplets in these cells. At the molecular level, the expression of the main adipogenes was significantly higher in mature adipocytes, and 17-β-estradiol increased the expression of these genes. In the same way, C/EBP alpha levels were also higher in the presence of β-estradiol (Figure 5d), although this effect was not obvious for the other proteins involved in adipocyte differentiation at least in this cellular model. These findings align with the results reported by Fatima et al. [26], who described a significant impact of β-estradiol on adipocyte differentiation, which is visible in the increase in adipogenes and proteins involved in this process. The suggestion that E2-induced effects on adipocyte differentiation may not involve PPAR-gamma activity [26] has also previously been postulated by authors such as Jeon and Yoong, who proposed a potentiation in estrogen deficiency states [27]. This could justify a lower presence of PPAR-gamma2 in our study, but not an increase in the expression of the *PPARG* gene. On the other hand, it is also possible to consider a low sensitivity of the Western blot technique.

The current study shows for the first time that the effects of β-estradiol on adipogenesis are altered by the presence of prelamin A. Increased immature farnesylated prelamin A reduced the number of lipid droplets and had different effects on gene expression, depending on whether total or farnesylated prelamin A was increased. However, combined treatment with β-estradiol and prelamin A maturation inhibitors revealed that the accumulation of farnesylated prelamin A significantly diminished the effect of β-estradiol on the main adipogenic gene expression and, at least, on the amount of C/EBP alpha protein levels.

The role of prelamin A accumulation in humans is not well defined. Thus, some studies [17] argue that murine farnesylated prelamin A accumulation causes progeroid syndromes, while non-farnesylated prelamin A is related to cardiomyopathy. However, there appears to be a more unanimous consensus that the abnormal accumulation of farnesylated prelamin A affects cellular division and proliferation rates [16].

In a prior study, we proposed that prelamin A accumulation may be necessary but not sufficient to induce the loss of adipose tissue [9]. Thus, in a patient with FPLD2, prelamin A accumulation was observed in adipose tissue from both lipoatrophic (thigh) and lipohypertrophic (neck) areas. The current study sheds light on why the onset of lipoatrophy occurs during the pubertal period in women, suggesting that the toxic effect of farnesylated prelamin A is conditioned by its ability to alter the action of estrogens on adipogenesis. On the other hand, our group did not find an accumulation of prelamin A, either total or farnesylated, in earlier studies involving cultured fibroblast from FPLD2 patients [28]. This apparent discrepancy is likely attributed to the type of cells studied, as the fibroblasts in the study by Tu et al. obviously came from the skin, which is unaffected in FPLD2, unlike progeroid laminopathies such as Hutchinson–Gilford Progeria Syndrome.

Recent studies [29] point to an overexpression of a micro-RNA, miR-355, which prevents the differentiation of preadipocytes into mature adipocytes, as a pathogenic mechanism of fat loss in FPLD2. Our study corroborates the involvement of prelamin A accumulation, suggesting complex interplay among multiple factors beyond prelamin A and estrogens.

This study has some main limitations. On the one hand, the use of 3T3-L1 cells for the study of adipogenesis is one of the most widely employed models today. While it is true that these models offer simplicity, there are other models that more accurately represent the complexity of diseases like FPLD2. Another important limitation of this study is that a murine cell model was used, in which the sex of the donors was not taken into account. Given the phenotypic sexual dimorphism of FPLD2 patients, our results should be interpreted with caution. Although one of the most efficient in vitro models involves the use of patient-derived cells with FPLD2 variants, obtaining preadipocytes from biopsies performed in the thigh of prepubescent women with *LMNA* pathogenic variants presents challenges. Recent studies, such as that by C. Xiao et al., propose the use of induced pluripotent stem cells (iPSCs) generated from peripheral blood mononuclear cells (PBMCs) of a patient with the p.R349W variant in the *LMNA* gene, although this option is not yet commercially available [30]. Finally, in vivo models provide a highly effective alternative for studying these diseases. Knock-out murine models for *LMNA* have been developed as well as models with point variants. However, they do not appear to mimic human disorders [31,32]. Since most *LMNA* variants seem to occur in exon 8, Wojtanik et al. created a murine model for R482Q. This model exhibited some phenotypic similarities to FPLD2, but differences were found in fat distribution between males and females, as observed in humans, but without prelamin A accumulation [33].

Although our results at the protein level were not conclusively robust, when taken together, the significant reduction in mature adipocytes, coupled with the evident decrease in the expression of the main adipogenes after estradiol and AFCMe treatment, supports our hypothesis.

From a clinical perspective, and with the aforementioned caution, these findings provide insights into the mechanisms triggering the phenotype onset in women with Dunnigan disease, typically occurring around puberty.

## 4. Materials and Methods

### 4.1. Adipose Tissue Biopsy and Cell Cultures

3T3-L1 murine preadipocytes were obtained from the European Collection of Cell Cultures and cultured on 35 mm multiwell dishes (6-well plates) in Dulbecco’s modified Eagle’s medium (DMEM; Sigma-Aldrich, St. Louis, MO, USA) plus 10% fetal bovine serum (FBS; Gibco, Carlsbad, CA, USA) and gentamicin 1.05 × 10^−4^ mol/L. For this study, 3T3-L1 cells between 10 and 15 passages were employed. On the other hand, a control adipose tissue sample was obtained from a non-lipodystrophic 6-year-old boy who had undergone programmed abdominal surgery for cryptorchidism, having obtained informed consent from his parents, which was in accordance with current Spanish legislation. The protocol was approved by the Ethics Review Panel of the Xunta de Galicia and carried out according to the ethical guidelines of the Helsinki Declaration. Small pieces of adipose tissue were placed on a 60 mm dish (BD Falcon^TM^; Mississauga, ON, Canada) containing DMEM plus 30% FBS and gentamicin 1.05 × 10^−4^ mol/L and then incubated at 37 °C with 5% CO_2_ in a Water-Jacket CO_2_ incubator (NuAire; Plymouth, MN, USA). Preadipocytes were recognized by the presence of small lipid droplets in the fibroblast-like cells. Subsequently, these preadipocytes were tripsinized (TrypLE™ Express Stable Trypsin-like Enzyme with Phenol Red; Gibco Life Technologies; Carlsbad, CA, USA) and cultured on 100 mm dishes in DMEM containing 10% FBS and gentamicin 1.05 × 10^−4^ mol/L.

### 4.2. Adipocyte Differentiation

After confluence, 3T3-L1 preadipocytes were induced to differentiate by incubation for 3 days in DMEM differentiation medium containing 10% FBS, insulin (1.74 × 10^−7^ mol/L), dexamethasone (2.5 × 10^−7^ mol/L), 3-isobutyl-1-methylxanthine (1 × 10^−4^ mol/L in DMSO), and a PPARG agonist: pioglitazone (1 × 10^−5^ mol/L in DMSO; Enzo Life Sciences, Lausen, Switzerland). Thereafter, incubation was carried out for an additional two days in growth medium containing insulin (1.74 × 10^−7^ mol/L) and pioglitazone (1 × 10^−5^ mol/L in DMSO) and excluding dexamethasone and 3-isobutyl-1-methylxanthine. Finally, cells were maintained for another two days in basal medium to attain more than 50% differentiated cells.

### 4.3. 17-β-Estradiol Treatment and Inhibition of Lamin A/C Maturation

The 3T3-L1 preadipocytes were induced for differentiation over eight days in the presence or absence of 17-β-estradiol (E2; 1 × 10^−9^ mol/L in DMSO; Sigma-Aldrich, St. Louis, MO, USA). Non-differentiated preadipocytes were supplemented with equivalent concentrations of DMSO and used as controls. Prelamin A accumulation was induced through the specific inhibition of lamin A precursor maturation steps. To accumulate non-farnesylated prelamin A, cells were cultured in the presence of the farnesyltransferase inhibitor 277 (FTI-277) (Calbiochem, San Diego, CA, USA), 2 × 10^−5^ mol/L for the first 24 h [34] and then 2.5 × 10^−6^ mol/L for the next seven days of differentiation. On the other hand, the accumulation of farnesylated carboxymethylated prelamin A was obtained using N-Acetyl-S-farnesyl-L-cysteine-methyl ester (AFCMe; Alexis Biochemicals, San Diego, CA, USA) [34,35] added to culture medium at the concentration of 1 × 10^−5^ mol/L for the first 24 h and then 1 × 10^−6^ mol/L for the next seven days of differentiation [36]. Hormones, FTI-277 and AFCMe were replaced every other day.

### 4.4. Oil Red O Staining

At day eight, after the induction of adipogenic differentiation, *Oil Red O* staining was used to reveal fully differentiated cells, which were washed with PBS and fixed in 10% formalin in isotonic buffer for 1 h at room temperature. After rinsing three times with double-distilled H_2_O to remove residual formalin, lipid vacuoles were stained with 0.6% *Oil Red O* (*w*/*v*) in 60% isopropanol for 1 h at 22 °C and washed three times with double-distilled H_2_O [35,37]. Following *Oil Red O* staining, the appearance of cytoplasmic lipid droplets was monitored by bright-field microscopy with an Olympus IX51 microscope (Olympus Corporation, Tokyo, Japan) and photographed with an Olympus DP72 digital (Olympus Life Science, Tokyo, Japan) camera using the cellSens software (Olympus Life Science, Tokyo, Japan, https://www.olympus-lifescience.com/en/software/cellsens/#!cms[focus]=cmsContent6016, accessed on 17 January 2024).

### 4.5. RNA Extraction and Retrotranscription

Total RNA was extracted from murine preadipocytes using Trizol (Invitrogen, Madrid, Spain) as per the manufacturer’s instructions. RNA was reverse-transcribed using M-MLV reverse transcriptase (Invitrogen, Madrid, Spain) as previously described [38].

### 4.6. Real-Time PCR

Specific primers and probes designed by Universal ProbeLibrary (Roche Diagnostics, Sant Cugat del Valles, Spain) were used in a Light Cycler 2.0 (Roche Diagnostics, Sant Cugat del Valles, Spain) to specifically determine the expression of the following adipogenic genes: *LMNA*, *PPARG*, *LPL*, *SLC2A4*, *CEBPA*, and *CEBPB* (Table 1). Real-time PCR conditions are available upon request. Results were normalized for the internal control *RN 18S* gene using the 2^−ΔΔ CT^ method [39].

### 4.7. Western Blotting

Whole-cell lysates were obtained from murine preadipocytes by sonication (Digital Sonifier with 102C CE Converter, Branson, MO, USA) in RIPA buffer (NaCl 0.015 mol/L, NP-40 1%, sodium deoxycholate 0.5%, SDS 0.1% and Tris-HCl 0.005 mol/L, pH 8) containing 5 × 10^−5^ mol/L DTT, 1 × 10^−4^ mol/L PMSF, 0.005 mol/L NaF, 1 × 10^−4^ mol/L Na_3_VO_4_ and a protease inhibitor cocktail (Sigma-Aldrich, St. Louis, MO, USA) at 4 °C. Two alternative protein extraction methods were used for the lamin A/C detection assays: (i) final pellets (insoluble material) obtained with the lysis buffer mentioned above were solubilized using hot (100 °C) loading buffer for 1 min; (ii) preadipocytes were lysed in ‘hot’ lysis buffer (90 °C) containing Tris–HCl 0.002 mol/L (pH 7.5), SDS 1%, Na_3_VO_4_ 1 × 10^−4^ mol/L, PMSF 1 × 10^−4^ mol/L, 2-mercaptoethanol 5% and protease inhibitors. After centrifugation (20,000× *g*, 15 min, 4 °C), 25–45 μg supernatant proteins were subjected to SDS gradient gel (8–12%) electrophoresis and transferred to a nitrocellulose (Amersham™ Hybond™-ECL, 0.45 μm, GE Healthcare, Piscataway, NJ, USA) or PVDF (Immobilon-P, 0.45 μm, Millipore, Bedford, MA, USA) membrane. Blots were blocked for 1 h at room temperature in 2% or 5% non-fat milk in PT (PBS, Tween-20 0.1%). Incubation with primary antibodies was performed for 1 h at room temperature (lamin A/C, PPAR-gamma2 and α-tubulin and GAPDH) or overnight at 4 °C (C/EBP alpha, C/EBP beta). The blots were probed with anti-PPAR-gamma2 diluted 1:200 (G-18, sc-22020, Santa Cruz Biotechnology Inc., Santa Cruz, CA, USA), anti-C/EBP beta diluted 1:500 (C-19, sc-150, Santa Cruz Biotechnology Inc., Santa Cruz, CA, USA), anti-C/EBP alpha diluted 1:1000 (p42, #2843, Cell Signaling Technology Inc., Danvers, MA, USA), anti-lamin A/C diluted 1:500 (N-18, sc-6215, Santa Cruz Biotechnology Inc., Santa Cruz, CA, USA). Monoclonal anti-α-tubulin diluted 1:20,000 (T5168, Sigma-Aldrich, St. Louis, MO, USA) and anti-GAPDH diluted 1:20,000 (G9545, Sigma-Aldrich, St. Louis, MO, USA) were used as loading controls. Anti-rabbit IgG peroxidase conjugated (#32460, Thermo Scientific, Rockford, IL, USA) or anti-mouse IgG peroxidase conjugated (NA931V, GE Healthcare, Piscataway, NJ, USA) were the secondary antibodies, which were both diluted 1:5000. Bands were revealed by chemiluminescence with the Supersignal West Dura extended duration substrate detection system (Thermo Scientific, Rockford, IL, USA). Subsequently, the bands obtained for each experiment (C/EBP beta, C/EBP alpha, PPAR-gamma2 and Lamin A/C) and condition were quantified separately and normalized to their corresponding loading control using the ImageJ software (release 1.53k, National Institutes of Health, Bethesda, MD, USA).

### 4.8. Immunofluorescence

The efficacy of FTI-277 and AFCMe treatments was checked by immunofluorescence in control human preadipocytes, as specific murine antibodies distinguishing between carboxymethyl-farnesylated and full-length non-farnesylated prelamin A are not available. Cells grown on glass coverslips were fixed with 4% paraformaldehyde at 4 °C for 1 h and permeabilized in 0.1% Triton X-100 at room temperature for 10 min. After blocking for non-specific binding (4% BSA, 1 h at room temperature), the coverslips were incubated overnight with the appropriate primary antibody at 4 °C (anti-full length prelamin A diluted 1:100 (ANT0045, Diatheva, Fano, Italy), anti-carboxymethyl-farnesylated prelamin A diluted 1:10 (ANT0046, Diatheva), anti-emerin diluted 1:50 (Novocastra, Leica, Barcelona, Spain), and anti-lamin A/C diluted 1:100 (N-18, Santa Cruz)). The ANT0045 does not bind carboxymethylated–farnesylated prelamin A, while no cross-reaction is observed between ANT0046 and full-length non-farnesylated prelamin A [6]. The following day, after three washes, the coverslips were incubated for 1 h at room temperature in the dark with Cy2-AffiniPure F(ab′)2 Fragment and Cy3-AffiniPure F(ab′)2 Fragment both diluted 1:600 (Jackson Immunoresearch, West Grove, PA, USA) and counterstained with 4,6-diamino-2-phenylindole DAPI diluted 1:1000 (Life Technologies, Madrid, Spain). Coverslips were mounted in Fluoromount medium (Sigma, Barcelona, Spain). Cells were visualized with an Olympus IX51 microscope (Olympus Corporation, Tokyo, Japan) and photographed with an Olympus DP72 digital camera using the cellSens software (Olympus Life Science, Tokyo, Japan, https://www.olympus-lifescience.com/en/software/cellsens/#!cms[focus]=cmsContent6016, accessed on 17 January 2024). All images were taken at similar exposures within an experiment for each antibody. Images were processed using ImageJ software (release 1.53k, National Institutes of Health, Bethesda, MD, USA).

### 4.9. Statistical Analysis

The statistical significance was determined using the non-parametric Kruskal–Wallis test, which was followed by a Mann–Whitney U post hoc test with Bonferroni correction. Real-time PCR analyses were performed in duplicate, n = 4. Data are presented as mean ± SD with statistical significance set at *p* < 0.05. All statistical analyses were performed using SPSS for PC (release 22.0; SPSS, Chicago, IL, USA).

## 5. Conclusions

In summary, our study aimed to explore the impact of 17-β-estradiol on murine 3T3-L1 preadipocyte adipogenesis in the context of prelamin A accumulation. Variations were noted based on β-estradiol exposure and the use of inhibitors (FTI-277 and AFCMe).

Prelamin A accumulation in nuclei, implicated in adipogenesis, was observed, which was in line with prior studies. Although farnesylated and non-farnesylated prelamin A were found in the nuclei, toxicity seemed to be associated with peripheral accumulation. However, this alone may not impair adipogenesis [7].

The well-established influence of sex hormones, particularly estrogen, on adipogenesis, and the involvement of various factors in preadipocyte differentiation were confirmed. The expected full adipocyte differentiation, increased lipid droplets, and elevated expression of main adipogenic genes, including C/EBP alpha with β-estradiol, were observed.

Interestingly, the effects of β-estradiol were altered by the presence of prelamin A. Farnesylated prelamin A reduced lipid droplets and influenced gene expression, but its accumulation hindered the impact of β-estradiol on adipogenic gene expression and C/EBP alpha protein levels.

The role of prelamin A in humans remains unclear, with studies proposing associations with progeroid syndromes and cardiomyopathy. Our study supports the notion that prelamin A accumulation may be necessary but not sufficient for adipose tissue loss.

Despite limitations in our experimental model, the reduction in mature adipocytes and decreased adipogenic gene expression after estradiol and AFCMe treatment support our hypothesis. In conclusion, our results suggest that the coexistence of 17-β-estradiol with farnesylated prelamin A accumulation impairs adipogenesis. Although these results obtained from a murine cell model should be taken with caution when extrapolating them to humans, they could provide an explanation for the fact that the loss of adipose tissue in Dunnigan’s disease becomes evident around puberty in women.

## Figures and Tables

**Table 1:** Primer sequences and probes.

	Accession Number NCBI	Forward Primer	Reverse Primer	Probe Number	Probe Sequence
LMNA	NM_019390	ATCCGCATTGACAGCCTCT	TCCAGGTCACGCAGCTTT	102	CTCAGCCA
PPARG	NM_011146.3	GAAAGACAACGGACAAATCACC	GGGGGTGATATGTTTGAACTTG	7	CTTCTCCC
LPL	NM_008509.2	GCTGGTGGGAAATGATGTG	TGGACGTTGTCTAGGGGGTA	25	CTCCTCCA
GLUT4 (SLC2A4)	NM_009204.2	GACGGACACTCCATCTGTTG	GCCACGATGGAGACATAGC	5	TGTGGCTG
Rn18s	NR_003278.2	AAACGGCTACCACATCCAAG	TACAGGGCCTCGAAAGAGTC	74	CTGCTGCC
C/EBPβ	NM_009883.3	TGATGCAATCCGGATCAA	CACGTGTGTTGCGTCAGTC	102	CTCAGCCA
C/EBPα	NM_007678.3	AAACAACGCAACGTGGAGA	GCGGTCATTGTCACTGGTC	67	TGCTGGAG

## Data Availability

Data are contained within the article.
